# A call to establish a child-centred disaster management framework in Zimbabwe

**DOI:** 10.4102/jamba.v7i1.148

**Published:** 2015-06-17

**Authors:** Ramphal M. Sillah

**Affiliations:** 1 Department of Development Studies, Midlands State University, Zimbabwe

## Abstract

Disasters have increased in intensity and frequency in recent times. However, disasters do not affect all groups in a society in a similar manner. This article, based mainly on qualitative desk research and document analysis, aims to illuminate the specific vulnerability of children to hazards and disasters. The research showed that owing to their special physiological, psychological, emotional and economic stature, children are an inherently vulnerable group. The paper advocates for existing disaster management structures and systems in Zimbabwe to elevate reduction of disaster risk amongst children within the scope of child protection, which aims to create a protective environment that shelters children from any form of harm or abuse. The paper proffers recommendations on how to design disaster management programmes in Zimbabwe with the needs of children in mind.

## Introduction

Disasters are occurring more regularly and with more intensity all over the world. Zimbabwe has been affected by various hazards and disasters in the past, particularly drought, floods, the HIV and/or AIDS pandemic, cholera outbreaks and transport accidents. It should be noted, however, that hazards do not necessarily transform into disasters; rather, they become disasters when they affect highly vulnerable communities with limited capacity to deal with the hazards. Essentially, hazards do not affect all groups in a society in a homogeneous manner. Different groups in a society have different levels of vulnerability to hazards and subsequent disasters owing to different political, social, economic and physical orientations. It would therefore be inadvisable to approach disasters with a view that the same kind of disaster risk reduction initiatives, disaster response ideals and disaster recovery ideals apply to every member of an affected community. For a country's disaster management practice to be as effective as possible, measures should be put in place to ensure that the specific vulnerabilities of all members of society are addressed.

Children are a particular group in society that needs unwarranted attention within the continuum of disaster management. In Zimbabwe, there is unfettered evidence that the government and other stakeholders acknowledge this group of people to be very vulnerable in most areas of human existence. The government has partnered with other non-state actors to draw up a commendable child protection system, which exudes legislation and policies aimed at protecting children, establish institutions aimed at identifying and protecting children at risk, with measures put in place to reduce their risk through legislation, and setting up a system aimed at assessing the effectiveness of these institutions and policies. Zimbabwe has been hailed in southern Africa for having a standalone act aimed at upholding children's rights, namely the Children's Act of 2001 (Zimbabwe 2001).

However, a weakness is that the child protection system in Zimbabwe largely conceptualises the need to protect children against child abuse. The system focuses on preventing child abuse in its physical, spiritual and emotional form, with emphasis specifically on sexual abuse. This focus is seen in practice by the growth of the Victim Friendly System in Zimbabwe, of which the major aim is to protect women and children from physical abuse, and sexual abuse in particular. It is estimated that there are 267 Victim Friendly Units in Zimbabwe (UNICEF 2010a). However, children's vulnerability with regard to hazards and disasters are not addressed by this system. Measures should be put in place to protect children from hazards and disasters, which can create unsafe environments for children. For this to happen, the vulnerabilities facing children in light of hazards and potential disasters need to be investigated and an appropriate, targeted disaster management mechanism needs to be set up. Investigating these vulnerabilities will increase the capacity of disaster managers to ensure that disaster management in Zimbabwe caters for the needs of a group so important in the development discourse.

## Conceptual framework

In Zimbabwe, a child is defined as anyone under the age of 18 years (Zimbabwe 2013); however, socio-cultural and economic connotations are also implied.

A disaster is defined as a:

serious disruption of the functioning of a community or a society involving widespread human, material or environmental losses and impacts, which exceeds the ability of the affected community to cope using its own resources. (UNISDR 2009:2)

From a technical point of view, however, disasters occur when hazards impact on highly vulnerable communities who have little capacity for dealing with them. A hazard refers to a dangerous phenomenon, substance, human activity or condition that may cause loss of life, injury or other health impacts, property damage, loss of livelihoods and services, social and economic disruption or environmental damage (Blaikie *et al.* 1994). Vulnerability is defined as the characteristics and circumstances of a community, system or asset that make it susceptible to the damaging effects of a hazard. Vulnerability is therefore a set of prevailing or consequential conditions arising from various physical, social, economic and environmental factors that increase the susceptibility of a community to the impact of hazards (UNISDR 2009). Vulnerability can also be seen as political, social, economic and physical aspects that affect a community's ability to respond to events (Jegillos 1999). It is this nature of vulnerability that renders different groups in a society susceptible to hazards in different ways. Capacity refers to the ability of people, organisations and systems to face and manage adverse conditions such as hazards and disasters using available resources (UNISDR 2009). Simple resources such as human knowledge, social relationships, leadership skills, physical strength and past disaster response experience give practical credence to the concept of capacity.

**FIGURE 1:**
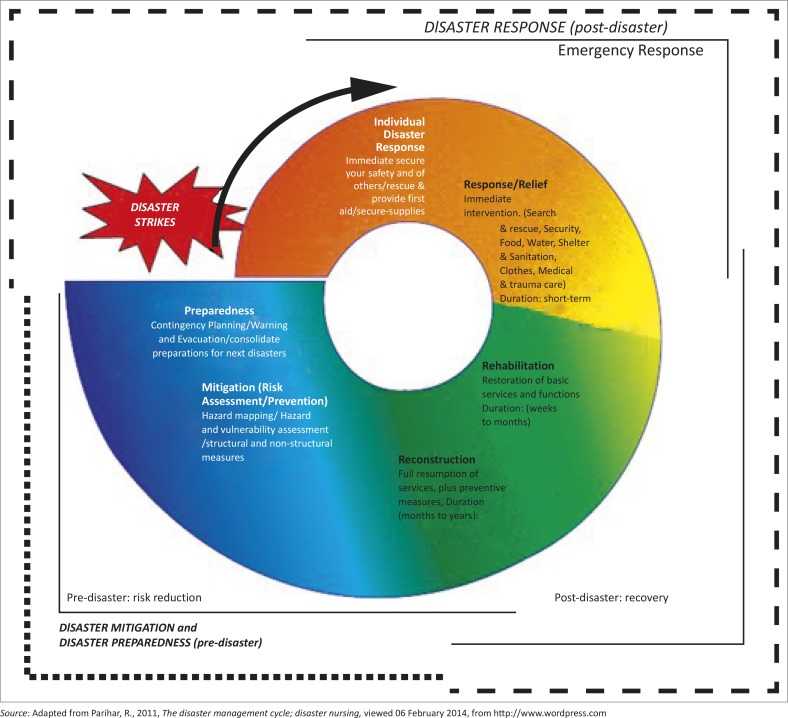
Disaster management cycle.

Disaster management refers to the systematic process of using administrative decisions, organisational and operational skills, and capacities to implement policies, strategies and coping capacities to lessen the impacts of a hazard. The major emphasis is on ensuring that a hazard situation does not escalate into a disaster. Disaster management thus involves putting in place efforts to prevent, mitigate, prepare for, respond to and recover from the impact of an adverse event (UNISDR 2009). In essence, disaster management starts with disaster risk reduction, which is defined as a conceptual framework of elements that takes into account the possibilities for minimising vulnerabilities and disaster risks to avoid or limit the adverse impacts of hazards, within the broad context of sustainable development (UNISDR 2009). This is followed by prompt response and recovery, which is largely guided by the thinking associated with reconstructing a damaged society, including disaster risk reduction elements. Disaster managers should therefore strive to reduce the vulnerability of a community to hazards whilst increasing a community's capacity to deal with hazards at the same time. An initial risk assessment is imperative to serve as a basis for improved disaster risk reduction to be put in place. Generally, the disaster management cycle has largely been used as a framework according to which disasters can be managed efficiently. A typical depiction of a disaster management cycle is shown in Figure 1.

Child-centred disaster management seems to be a combination of disaster management and children's rights. In Africa, children's rights are guided mainly by the *African Charter on the Rights and Welfare of the Child* (African Union 1999). Children's rights can be considered according to the so-called three P's: child protection, child participation and provision. As child protection has been defined earlier in the manuscript, it is not necessary to be redefined in the context of children's rights. Child participation is the process of actively involving children in issues that affect them. It is about ensuring that children are integral players in the design, implementation, monitoring and evaluation of initiatives that affect them. Provision refers to ensuring that children have access to basic needs such as shelter, food, health care and education (REPSSI 2004).

It should be noted that there is no international definition of child-centred disaster management, as this is a new and undersubscribed area. Ideally, child-centred disaster management is about identifying child-specific risks and putting measures in place to ensure that children's needs are observed and upheld throughout the disaster management cycle. It is about planning and designing disaster management programmes with child-centred vulnerabilities and the holistic notion of children's rights in mind. Child-centred disaster management initiatives should be developed in collaboration with children, thereby upholding their right of participation. In addition, it is about rectifying the discord between children's rights and the effects of hazards and subsequent disasters to ensure the physical, emotional, spiritual and psychological development of children. In short, child-centred disaster management is about making sure that the three P's are met throughout the disaster management cycle, so that children are protected, are provided for and can participate in disaster management.

## Methodology

The paper aims to advocate for the institution of child-centred disaster management in Zimbabwe. The proposed framework was developed based mainly on desktop research, with an emphasis on discourse analysis. A reasonable number of texts and documents on disaster management in Zimbabwe were analysed. Texts with a substantial bearing on child protection and disaster management policy were interrogated and analysed with a view towards critiquing the truth within these texts (Burr 1995). The major question addressed in this study was to assess to what extent the disaster management framework in Zimbabwe upholds and protects the rights of children. Suggestions for initiatives that can make the disaster management framework in Zimbabwe more child friendly are based on the outcome of the research.

## Hazards and disasters in Zimbabwe: A child-centred hazard analysis

There is a dire need to justify the fact that children are affected by hazards and disasters in Zimbabwe. Zimbabwe is mainly vulnerable to the effects of floods, drought, the HIV and/or AIDS pandemic, cholera outbreaks and transport accidents. Between 1980 and 2010, 35 natural disasters have been recorded, resulting in 6448 deaths (PreventionWeb 2012). This translates to an average of 208 deaths per year due to natural disasters. These 35 natural disasters included six droughts, seven floods, two cyclones and 20 occurences of epidemics (PreventionWeb 2012).

Drought and famine have occurred regularly in Zimbabwe. The worst drought in Zimbabwe was that of 1991/1992, during which five million people were affected by food insecurity (PreventionWeb 2012). Children are directly affected owing to their inherent need for proper nutrition to support physical development. It is estimated that 11% of children are born with low birth weight, with 10% of children younger than 5 years being moderately underweight, 2% being severely underweight and 32% presenting with signs of stunted growth (UNICEF 2010b). Similarly, the World Food Programme has reported a 34% stunting rate amongst Zimbabwean children younger than 5 years (World Food Programme 2012). These statistics invariably show that children are affected by drought and famine.

The HIV and/or AIDS pandemic has wreaked havoc in Zimbabwe. It is estimated that one in every five Zimbabweans lives with HIV and/or AIDS (USAID 2011). In 1998, Zimbabwe reported the third highest HIV prevalence in the world at 33%. However, the current prevalence is estimated at 14.6% (Index Mundi 2013). Children have not been spared from the epidemic. Approximately 35 000 children are in need of antiretroviral therapy, with only 17 000 accessing the treatment at the moment (UNICEF 2010b). HIV and/or AIDS has adversely affected children also in indirect ways, as seen in the phenomenon of child-headed households, thereby compromising the cross-section of provision rights. According to Bongo *et al.* (2013) approximately 600 000 children have been orphaned owing to the effects of AIDS. The HIV and/or AIDS pandemic can therefore be considered to have created an environment of risk amongst children.

Transport accidents have adversely affected Zimbabwean children as well. An example is the Nyanga bus disaster, in which 89 people, mostly children going on a field trip, died in 1991. Similar examples include a boat capsizing in Lake Chivero in 1995, killing 22 children, and another in 2011, during which 11 children died (Chikoto & Sadiq 2012).

## Vulnerability of children to hazards and disasters in Zimbabwe: A theoretical analysis

The brief snapshot of hazards and disasters in Zimbabwe shows that children are indeed directly affected by hazards and disasters. There is an inherent need to determine what makes children susceptible to various hazards and subsequent disasters. Such an assessment could provide the basis for possible disaster risk reduction initiatives in future. This section aims to investigate these aspects of vulnerability amongst children theoretically and interrogates aspects that are likely responsible for exposing children to hazards and subsequent disasters.

Physical vulnerability is largely associated with aspects of location and levels of mobility in relation to the hazard. Children are often in buildings or environments that pose great risk to hazards and subsequent disasters. An applicable example of such an environment is a school, where a high concentration of children in a single location at the same time constitutes a high risk. A large number of people concentrated in one area are associated with various risks, such as the risk of increased spread of contagious diseases such as cholera, measles and chicken pox, for example. Most of the schooling infrastructure in Zimbabwe, particularly in rural areas, was inherited from the colonial system. Water and sanitation systems are dated and no longer meet the desired standards, thereby exposing children to cholera, for example (UNDP 2010). The building materials have also been eroded, which makes collapse in the face of hazards such as cyclones, floods and earthquakes possible.

There is also the possibility of sociological hazards being inflicted upon children at schools, because the large gathering of people in one place makes the school a viable target for terrorism and other sociological hazards. An example is the Sandy Hook Elementary School disaster in Connecticut, USA, where 20 students and 6 adults were killed (CNN 2013). To this end, children, by virtue of going to school, are exposed to various hazards and possible disasters. Another example of hazards associated with human negligence or terrorism that may affect children comes from an incident in 2013 in which 23 students in the Saran District, India, died after having eaten a school meal laced with pesticide (Reuters 2013).

During times of emergency, physical mobility is an essential attribute of disaster response. As much as emergency services have the task of rescuing people, their efficiency is also based on the ability of the people in need to work with the rescuers. The ability to climb a tree in a flooded area, hold onto a rescue rope or swim towards a rescue boat can be defining actions in the face of emergency. Young children, however, often simply do not have the physical strength to help the rescuers help them. An example can be found in the Lake Chivero disasters mentioned earlier. The strength needed to swim towards shore or at least stay afloat until the rescue teams arrived was clearly lacking. Infants in particular are not mobile at all and rely on adults for their mobility. Thus, in the face of a hazard, the infant cannot put in place any self-help mechanisms to rescue themselves or contribute to their rescue. Evidently, emergency systems and resources in Zimbabwe have largely been designed to cater for adults and are not designed to cater specifically for children.

Owing to their limited physical strength, children are easy to abuse physically, and sexually in particular. During the chaos of calamity, girl children are particularly at risk of sexual abuse. In the aftermath of Hurricane Katrina, a surge in sexual crimes was realised. According to the National Sexual Violence Resource Centre, a national database funded by the Centre for Disease Control and Prevention to track sexual assaults that occurred after Hurricane Katrina, 42 reports of sexual assaults were received within 6 weeks of the website being launched (Burnett 2005). This suggests an inherent link between sexual violence, particularly against women, and disasters (Burnett 2005).

In Zimbabwe, children have, to a large extent, been ostracised from participating meaningfully in political matters of significance. Governance structures in Zimbabwe do not provide for the involvement of children in decision-making capacities. It should be noted that disaster risk reduction measures are propounded and sanctioned by political institutions such as, for example, local governments. Children are limited to participating in mainly ceremonial institutions such as child councils, child parliament and child protection committees, which do not have any significant effect on decisions being made. In Zimbabwean culture, society requires children to respect their elders, particularly at public gatherings. This ultimately suppresses the voice of the child members of the child protection committees, which consist of both adults and children. To this end, it is evident that children do not participate fully in political matters, thus rendering them extremely vulnerable with regard to disaster management.

From a social perspective, psychological immaturity, lack of education, lack of exposure to past disasters and limited life experience contribute to the vulnerability of children in the face of hazards and disasters. Limited life experience means that children have little knowledge about the cause and subsequent effects of disasters and might expose themselves unwittingly to various hazards. This lack of exposure can be compounded by the lack of education, which is a crucial determinant in mitigating exposure to hazards such as, for example, HIV and/or AIDS. Lack of adequate education can hinder an individual from amicably articulating disaster preparedness ideals such as early warnings and disaster preparedness plans. Past exposure to hazards and disasters can increase an individual's capacity to face hazards, but children have very little life experience.

Religious and cultural customs can also expose children to various hazards. In most cases such religious and cultural customs are dogmatic. Customs such as child marriage, child betrothal, female genital mutilation and male circumcision can expose children to HIV infection. Religious beliefs such as those opposed to the use of western medicine may have contributed to the prevalence of measles remaining a problematic biological hazard in Zimbabwe. By 2010, measles accounted for 1% of deaths in children younger than 5 years in Zimbabwe, which experts suggested could be attributed to the religious belief that denies children the right to immunisation (UNICEF 2010b). An ironic scenario in Zimbabwe has been that dogmatic cultural and social customs have been used as a way of mitigating against the harsh effects of hazards such as droughts. As a mitigation measure, girl children are married off in exchange for food. This might reduce the vulnerability of the family to drought, but consequently increases the girl child's vulnerability to a hazard such as HIV infection.

Socially, children are considered a bottom caste group in traditional Zimbabwean culture (Gil 1998). Children are considered last when it comes to entitlements. For example, when it comes to food, children are the last to eat or receive the smallest portions. This predicts an unfavourable scenario for children when it comes to disaster relief efforts, as children might be catered for last during the distribution of relief aid.

Children rely on adults for psychological and social stability. Adults form an indivisible psychosocial support system for children, which, when disturbed, leads to psychosocial distress on the part of children. However, during hazards and possible disasters, there is real risk that parents and guardians may succumb, leaving children in distress.

Children are inherently dependent upon their parents and guardians for economic sustenance, to the extent that children experience a form of ‘transient poverty’, described as a temporary consumption poverty at household level, which is directly attributable to variability in consumption (FAO 2013). It should be noted, however, that poverty is an underlying cause of vulnerability and an incremental effect that eventually gives rise to unsafe conditions. Children being ‘poor’ inherently means that they are a vulnerable group in the disaster management discourse.

## Towards effective child-centred management in Zimbabwe: Recommendations

It is evident that children are vulnerable to hazards and subsequent disasters in various ways. There is a definite need for disaster managers to design their disaster management programmes in a way that will encompass (mainstream) the needs of children and essentially reduce their vulnerability. This section will proffer suggestions to this end using the general disaster management cycle as framework.

### Disaster prevention and mitigation

Disaster prevention and mitigation is the first stage of the disaster management cycle. This stage involves putting in place structural and non-structural measures aimed at the direct avoidance of disaster as well as trying to reduce the possibility of hazards transforming into disasters. This process is referred to as disaster risk reduction (DRR) (UNISDR 2009).

Risk assessment should be the first step in establishing child-centred disaster prevention and mitigation. Essentially, all disaster managers undertake risk assessments in communities at risk, but it is fair to state that there is a tendency to treat all members of society as being homogeneous during the assessment. Instead, disaster managers should design risk assessment tools to produce child-disaggregated data. Specific attention should be given to assessing risks present in child-centred institutions such as schools. This will serve as a basis for investigating child-related vulnerabilities and contribute to designing relevant child-inclusive DRR initiatives.

The next aspect is to design ways for including children in developing DRR policies and initiatives. Examples include starting DRR committees for children, setting up DRR clubs at schools, or even including disaster management as part of existing peer education curricula. This will enhance and facilitate the participation of children in drawing up DRR measures that are aimed at serving them.

The major role of the DRR institutions for children should be to increase awareness about the cause-and-effect scenarios of disasters and provide mitigatory knowledge and exposure. To this end, the institutions must be instrumental in lobbying for disaster management to be part of the school curriculum. This is an ideal that has been adopted in India (Parihar 2011). The DRR institutions should also be responsible for training children on how to provide basic mitigation ideals, such as basic first aid or how to use fire extinguishers.

### Disaster preparedness

Disaster preparedness involves putting in place measures that allow people to react in the face of a disaster. Preparation ideals such as early warning systems, contingency plans, resource bases for disasters and training of communities on how to react in the event of a hazard are evident in typical disaster preparedness programming (UNISDR 2007).

Child-centred disaster preparedness should start with a disaster preparedness plan. Disaster preparedness plans should be presented in a manner that is child friendly and easy to understand. Disaster managers can use cartoons, road shows and songs, for example, to raise awareness amongst children on how to react in response to an early warning. This should be combined with routine simulation drills, particularly at child-centred institutions such as schools. In the event of a hazard occurring, children's lives may be saved because they would already have been exposed to simulated emergency situations and will therefore be more likely to know how to react.

As disaster managers identify and prepare for response and relief activities, a number of child-centred issues should be considered. The first aspect is the initial assessment. Disaster managers should ensure that the initial assessments produce child-disaggregated data. There is an inherent need to collect statistics on children affected as a result of the hazard or disaster. As disaster managers plan and prepare for response activities they should make it policy that women and children will be given priority during response activities such as search and rescue efforts.

Provision must be made for child-centred considerations. The idea is that during response and relief efforts, children's rights must consistently be observed. Relevant child-centred items such as vaccines and appropriate food stuffs such as powdered milk or fortification items should be budgeted for or stockpiled. Non-food items such as clothing, blankets, towels and napkins should also be included as part of the resource base. Disaster managers should also make provision for tents or temporary housing equipment that would be appropriate for serving as a temporary school facility. The aim is to ensure that children's rights are observed consistently. Furthermore, disaster managers should ensure that their human resource base comprises staff who can cater for the various needs of children. To this end, professionals such as teachers, counsellors and early-childhood development specialists should be part of the disaster response and relief contingent.

An important aspect to plan and prepare for is the provision of security at evacuation or site camps. There is a correlation between the occurrence of hazards and an increase in sexual abuse of women and girl children. To this end, security must always be available at site camps, so as to ensure that law and order is observed.

### Disaster response and relief

Disaster response and relief refers to the process of instituting immediate intervention in the aftermath of a disaster. It involves putting in place measures to save as many lives as possible and trying to reduce human suffering caused by the disaster. Facilitating access to basic human needs for communities in distress is key during this stage (UNISDR 2007).

During disaster response and relief, women and children should be given priority and disaster managers should adhere to the SPHERE standards. The SPHERE Project's *Handbook* (SPHERE Project 2011) is an internationally recognised handbook that sets out standards on how humanitarian agencies and aid workers should respond to disasters. The handbook consists of three documents, namely the Humanitarian Charter, the Humanitarian Worker Code of Conduct and the SPHERE Minimum Standards for Technical Areas (SPHERE Handbook 2011). The benefit of adhering to the SPHERE standards is that they are by nature sensitive to the needs of vulnerable groups and acknowledge the need to cater for groups such as children. Adhering to the SPHERE standards has the positive effect of counteracting traditional ideals, which regard children as a bottom caste group and subsequently means that they are usually the last to receive entitlements such as food. However, if minimum standards for all technical areas are adhered to everyone will be fully catered for. For example, the minimum standard for cereals is 10 kg per person per month. Therefore, if a mother has three children in the site camp, it automatically means that the household is entitled to 40 kg of cereal per month, which is a sufficient ration for all.

Disaster managers should endeavour to involve children during response and relief efforts. The roles that children are given during this phase of disaster management must be conversant with their age. Monitoring and evaluation of the disaster response and relief effort should be in conjunction with children. Their voices must be heard, as they are direct recipients of the disaster response and relief effort.

### Rehabilitation and reconstruction

Rehabilitation and reconstruction are sometimes referred to as the recovery phase of the disaster management cycle. Rehabilitation involves the process of resuming the normal functions and services of a community in the aftermath of a disaster. During this stage, stopgap measures are put in place to cover the losses experienced by the community as a result of the disaster. Re–opening of markets and removal of debris on roads after an earthquake are examples of activities undertaken during the process of rehabilitation. Rehabilitation is seen as a step towards more permanent reconstruction. Reconstruction involves the full resumption of services and rebuilding of infrastructural and institutional ideals. The idea is to put in place preventive measures aimed at making the community better equipped to deal with future hazards (UNISDR 2007).

Key child-appropriate initiatives should be considered during rehabilitation. As stated before, the aim is to ensure that children's rights are observed throughout the disaster management cycle. Disaster managers should put in place measures to ensure that birth certificates are re-issued. The obvious assumption is that in the aftermath of a calamity, such documents are destroyed. In addition, disaster managers should ensure that the families of children who arrive at site camps unaccompanied be traced. If the immediate relatives or other guardians of such children cannot be found, disaster managers should work with social workers to ensure that such children get access to alternative care.

As part of the rehabilitation process, education activities must continue. Education is therapeutic in nature and can help children process the losses incurred as a result of the disaster. Continued counselling is important as it can help to ease the psychological distress caused by the disaster.

As the cycle reaches the reconstruction stage, strengthening of capacities for child-centred disaster management is needed at all levels. Institutions should be redesigned with the possibility of ensuring that children participate in DRR initiatives. Child-centred facilities such as schools should be reconstructed with the aim of reducing risk. For example, after a school has been gutted by fire, the use of fire-resistant materials should be considered during reconstruction. Child protection systems should also be strengthened, with the aim of fostering child protection values to be enshrined in society even during times of distress.

## Conclusion

Children are the future of development; without them society will die. There is, therefore, an inherent need to protect children from all forms of harm, including disasters. The child protection system in Zimbabwe has neglected the protection of children from hazards and disasters. Children are vulnerable to hazards and subsequent disasters. In a bid to resolve these vulnerabilities, the child protection system in Zimbabwe needs to be remodelled to institute child-friendly measures throughout the disaster management cycle. The paper has described various aspects of vulnerability related to children and has proffered measures that can contribute to disaster management becoming more child friendly. A child-centred disaster management framework that is part of the overall child protection system in Zimbabwe is required. Without it, children remain vulnerable and exposed to harm.
